# Expression and Activity of COX-1 and COX-2 in *Acanthamoeba* sp.-Infected Lungs According to the Host Immunological Status

**DOI:** 10.3390/ijms19010121

**Published:** 2018-01-02

**Authors:** Natalia Łanocha-Arendarczyk, Irena Baranowska-Bosiacka, Karolina Kot, Izabela Gutowska, Agnieszka Kolasa-Wołosiuk, Dariusz Chlubek, Danuta Kosik-Bogacka

**Affiliations:** 1 Department of Biology and Medical Parasitology, Pomeranian Medical University in Szczecin, 70-204 Szczecin, Poland; nlanocha@pum.edu.pl (N.Ł.-A.); kotkarolina17@gmail.com (K.K.); 2 Department of Biochemistry and Medical Chemistry, Pomeranian Medical University in Szczecin, 70-204 Szczecin, Poland; ika@pum.edu.pl (I.B.-B.); dchlubek@pum.edu.pl (D.C.); 3 Department of Biochemistry and Human Nutrition, Pomeranian Medical University in Szczecin, 70-204 Szczecin, Poland; izagut@poczta.onet.pl; 4 Department of Histology and Embryology, Pomeranian Medical University in Szczecin, 70-204 Szczecin, Poland; agnieszka.kolasa@pum.edu.pl

**Keywords:** *Acanthamoeba* sp., lungs, cyclooxygenase 1 (COX-1), cyclooxygenase 2 (COX-2), prostaglandin E_2_ (PGE_2_), thromboxane B_2_ (TXB_2_)

## Abstract

Little is known about the pathomechanism of pulmonary infections caused by *Acanthamoeba* sp. Therefore, the aim of this study was to determine whether *Acanthamoeba* sp. may affect the expression and activity of cyclooxygenase-1 (COX-1) and cyclooxygenase-2 (COX-2), resulting in the altered levels of their main products, prostaglandins (PGE_2_) and thromboxane B_2_ (TXB_2_), in lungs of immunocompetent or immunosuppressed hosts. *Acanthamoeba* sp. induced a strong expression of COX-1 and COX-2 proteins in the lungs of immunocompetent mice, which, however, did not result in significant differences in the expression of PGE_2_ and TXB_2_. Our immunohistochemical analysis showed that immunosuppression induced by glucocorticoids in *Acanthamoeba* sp.-infected mice caused a decrease in COX-1 and COX-2 (not at the beginning of infection) in lung tissue. These results suggest that similar to COX-2, COX-1 is an important mediator of the pathophysiology in experimental pulmonary acanthamoebiasis. We suggest that the signaling pathways important for *Acanthamoeba* sp. induction of lung infection might interact with each other and depend on the host immune status.

## 1. Introduction

Free-living amoeba of the genera *Acanthamoeba* can infect the central nervous system, causing granulomatous amebic encephalitis (GAE) in both immunocompetent and immunosuppressed patients [1]. They also cause *Acanthamoeba* keratitis (AK), cutaneous acanthamoebiasis (CA), and lung infection (*Acanthamoeba* pneumonia) [2,3]. Park et al. [4] found that *Acanthamoeba* sp. can induce airway inflammation via a protease allergen. Pneumonia caused by *Acanthamoeba* sp. has also been reported in a child with congenital immunodeficiency and in a lung transplant patient [5,6].

The diagnosis of pulmonary infections with *Acanthamoeba* can be based on the identification of stages (trophozoites and/or cysts) in bronchoalveolar lavage samples and cultivations [7]. Our previous study was the first to isolate *Acanthamoeba* sp. in the bronchoaspirate of immunosuppressed patients with atypical symptoms of pneumonia [2]. In experimental animal models, *Acanthamoeba* sp. has been observed to cause damage to the pulmonary parenchyma, inflammatory infiltrate and destruction of the respiratory epithelium, blood vessels, bronchi and bronchioles [7,8].

The intensity of inflammation, e.g., in lung tissues, depends on the functionality of the immune system. In pneumonia, the innate immunity of the lung tissue may be regulated by the increased expression of cytokines, chemokines and cyclooxygenase (COX) [9,10]. In this paper, we focus on COX, the key enzyme in the conversion of arachidonic acid to prostaglandin (PGE), the precursor of bioactive lipid mediators, including PGs, thromboxanes (TXs) and prostacyclin. Prostaglandin E_2_ (PGE_2_) is the most important prostaglandin which mediates the typical symptoms of inflammation: rubor, calor, tumor and dolor. It may act on neurons and contributes to the systemic responses to inflammation such as fever and fatigue [11,12]. PGE_2_ also has a bronchodilatory effect and inhibits both early and late phase responses to allergens and other triggers of bronchoconstriction [13,14,15]. Thromboxanes are important mediators of inflammation. Thromboxane B_2_ (TXB_2_) is a metabolic product of thromboxane A_2_, a potent bronchoconstrictor [16]. Increased TXB_2_ levels have been reported in the airways of asthmatics following exposure to allergens [14,17].

Cyclooxygenase occurs in two isoforms, constitutive cyclooxygenase-1 (COX-1) and induced cyclooxygenase-2 (COX-2), which differ in their pattern of expression. In most tissues, COX-1 is involved in the maintenance of homeostasis, with increased expression in rheumatoid arthritis. The presence of COX-1 has been demonstrated in both the upper and lower respiratory tracts as well as in the pleura [18,19]. COX-2 participates in physiological and pathological processes, and its physiological presence has been identified. In the postnatal period, expression of COX-2 is low and limited to the bronchial epithelium and pulmonary alveoli [19,20]. COX-2 expression has been reported to be associated with inflammation or proliferative changes in the airway epithelium, including pneumonia and lung cancer [10]. Similar to COX-1, COX-2 plays an important role in allergic lung inflammation and asthma [9]. COX-2 has been shown to be induced by parasitic infections, but the mechanism is not clear [21]. In opportunistic parasitic infection, a strong correlation has been observed between COX-2 expression and *Cryptosporidium* sp. infection in the lungs and epithelial lining of the respiratory tract [22].

An immunoregulatory role for prostaglandins has been observed in *Entamoeba histolytica* and *Leishmania amazonensis* infections [23,24,25]. *Leishmania donovani* infections induce PGE_2_ generation within the host macrophages to aid in parasite survival [25]. PGE_2_ mediates immunosuppression that occurs in the acute phase of Chagas disease [26,27,28]. Anyona et al. [29] noted that the down regulation of PGE_2_ levels in *Plasmodium falciparum* infection correlated with increased clinical manifestation in brain *Plasmodium* infection, anemia and malaria during pregnancy. There is some evidence of a relationship between *Acanthamoeba* sp. infection and changes in PGF_2_ alpha depending on the pathogenicity of the amoeba strain. The pathogenic strains of *Acanthamoeba castellanii* produce more PGF_2_ alpha than non-pathogenic strains, but they do not differ markedly in PGE_2_ synthesis [30].

Understanding the mechanisms of acanthamoebiasis and how prostanoids modulate the immune response may help in limiting cellular damage, resulting in a development or change in pharmacological therapy. No studies to date have addressed the implications of the role of COX-1 and COX-2 enzymes and their metabolites in *Acanthamoeba* sp. lung infections in immunocompetent or immunosuppressed hosts. Thus, to clarify the role of prostanoids in lung acanthamoebiasis in relation to the host immune status, the purpose of this study was to determine whether *Acanthamoeba* sp. may affect the expression and activity of COX-1 and COX-2, resulting in altered levels of their main products, PGE_2_ and TXB_2_.

## 2. Results

### 2.1. Body Weight, Lung Weight and Lung Weight Ratio

**Figure 1:**
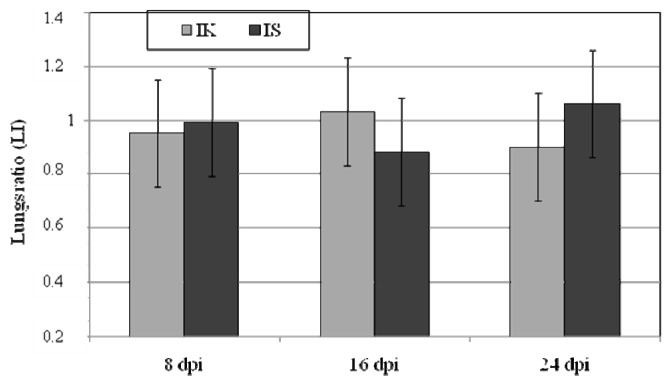
The relative weight ratio of lungs (LI) in relation to control groups: immunocompetent (IK) and immunosuppressed (IS) in various (8, 16, 24) days post *Acanthamoeba* sp. infection (dpi). Data represent the mean ± standard deviation (SD).

The body and lung weights of the mice in the control and infected groups are shown in Table 1, lung weight ratio is presented in Figure 1. We observed lower total body and lung weights in the immunocompetent *Acanthamoeba* sp.-infected mice compared to the immunocompetent control mice, and significant differences in total lung mass between mice from group A and the AS (Table 1). Statistically significant differences of total body mass were found between groups A and C and groups A and AS at 16 days post *Acanthamoeba* sp. infection (dpi). There were no statistically significant differences in lung weight at 8, 16 and 24 dpi in all groups. In immunocompetent *Acanthamoeba* sp.-infected mice, lung weight ratio was the highest at 16 dpi and in immunosuppressed infected mice at 24 dpi (Figure 1). Strain AM 22 showed pneumophilic properties; numerous amoebae trophozoites were observed 12 h after mice inoculation near the lung fragments from *Acanthamoeba* sp.-infected immunocompetent and immunosuppressed mice.

### 2.2. Acanthamoeba sp. COX-1 Expression in Lungs During Infection

**Figure 2:**
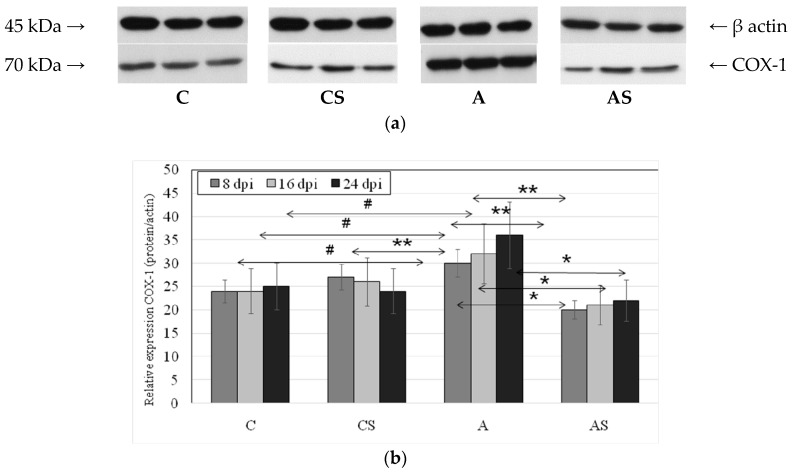
The effect of *Acanthamoeba* sp. infection on the expression of cyclooxygenase 1 (COX-1) in mouse lungs. Cyclooxygenase 1 was determined using Western blot analysis. Representative Western blots (**a**) and densitometric analysis of COX-1 protein normalized to β-actin (**b**) in mouse lungs were shown. Data represent the means± standard deviation (SD) for six in dependent experiments. # *p* < 0.05 vs .control group; * *p* < 0.05 infected immunocompetent mice vs. infected immunosuppressed mice, ** *p* < 0.05 vs. infected group, using a Mann–Whitney *U* test.

We observed statistically significant differences in lung COX-1 protein expression in *Acanthamoeba* sp.-infected immunocompetent mice vs. the immunocompetent control group (Figure 2). The highest expression of COX-1 protein in the lungs was found in *Acanthamoeba* sp.-infected immunocompetent mice at 24 dpi, significantly higher than in the control group (by 44%, *p* = 0.025). A significant increase in expression of the enzyme compared with control was also observed in group A at 16 dpi (33%, *p* = 0.022) and 8 dpi (12%, *p* = 0.042) vs. control group. Expression of the enzyme in group A was also significantly higher than in the *Acanthamoeba* sp.-infected immunosuppressed mice at 8 dpi (by 50%, *p* = 0.004), 16 dpi (by 52%, *p* = 0.002), and 24 dpi (by 64%, *p* = 0.002), and significantly positively correlated with the time of infection (rs = +0.49).

### 2.3. Acanthamoeba sp. COX-2 Expression in Lungs During Infection

**Figure 3:**
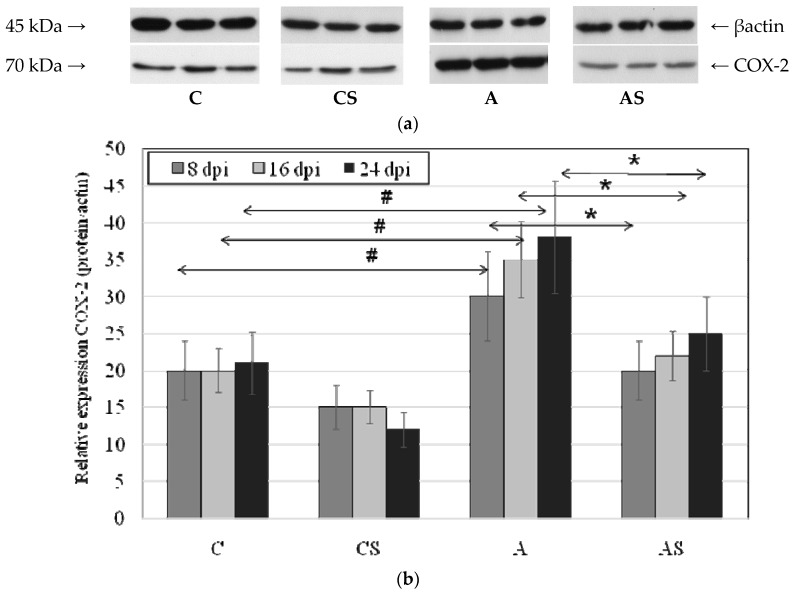
The effect of *Acanthamoeba* sp. infection on the expression of cyclooxygenase 2 (COX-2) in mouse lungs. Cyclooxygenase 2 was determined using Western blot analysis. Representative Western blots (**a**) and densitometric analysis of COX-2 protein normalized to β-actin (**b**) in mouse lungs were shown. Data represent the means ± standard deviation (SD) for six independent experiments. # *p* < 0.05 vs. control group; * *p* < 0.05 infected immunocompetent mice vs. infected immunosuppressed mice, using a Mann–Whitney *U* test.

In the *Acanthamoeba* sp.-infected immunocompetent mice the expression of COX-2 protein was significantly higher than in the control group at 8 dpi (by 50%, *p* = 0.002), 16 dpi (by 75%, *p* = 0.001) and 24 dpi (by 80%, *p* = 0.003). There was an upward trend in the expression of the enzyme in relation to the duration of infection, but the observed differences were not statistically significant. COX-2 expression was different in *Acanthamoeba* sp.-infected immunocompetent mice and was significantly higher than in *Acanthamoeba* sp.-infected immunosuppressed mice (Figure 3). The highest expression of the enzyme was observed in A group at 24 dpi, 52% higher (*p* = 0.003) vs. AS group. Also at 8 dpi COX-2 expression was 50% higher (*p* = 0.001) and at 16 dpi was significantly higher (by 59%, *p* = 0.004) compared to *Acanthamoeba* sp.-infected immunosuppressed mice.

### 2.4. PGE_2_ in Lungs During Acanthamoeba sp. Infection

**Figure 4:**
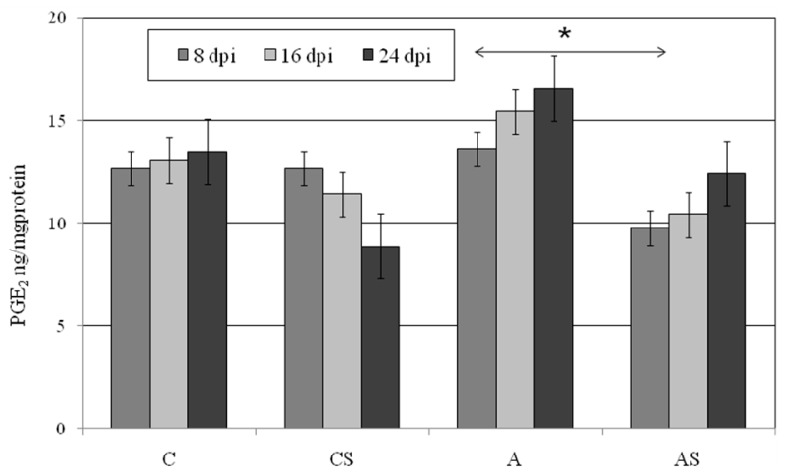
Changes in prostaglandin E_2_ (PGE_2_) levels in the lungs of *Acanthamoeba* sp.-infected mice depending on the host immune status (C, immunocompetent uninfected control group mice; CS, immunosuppressed uninfected mice; A, immunocompetent *Acanthamoeba* sp.-infected mice; AS, immunosuppressed *Acanthamoeba* sp.-infected mice; Data represent the mean ± standard deviation (SD) for six in dependent experiments * *p* ≤ 0.05 for the significance of difference A vs. AS (Mann–Whitney test; dpi days post infection).

We did not observe statistically significant differences in lung PGE_2_ concentration between the immunocompetent control group and *Acanthamoeba* sp.-infected immunocompetent mice (Figure 4). Lung PGE_2_ was the highest at 24 dpi in *Acanthamoeba* sp.-infected animals and almost 19% higher than in the uninfected control mice. In A group we observed an increase PGE_2_ concentration progressing with the duration of infection, but it was not statistically significant. There were also no statistically differences in lung PGE_2_ concentrations between AS and CS groups. We observed higher levels of lung PGE_2_ in mice from A group in comparison to mice from AS group at 8 dpi (*p* = 0.04).

### 2.5. TXB_2_ in Lungs during Acanthamoeba sp. Infection

**Figure 5:**
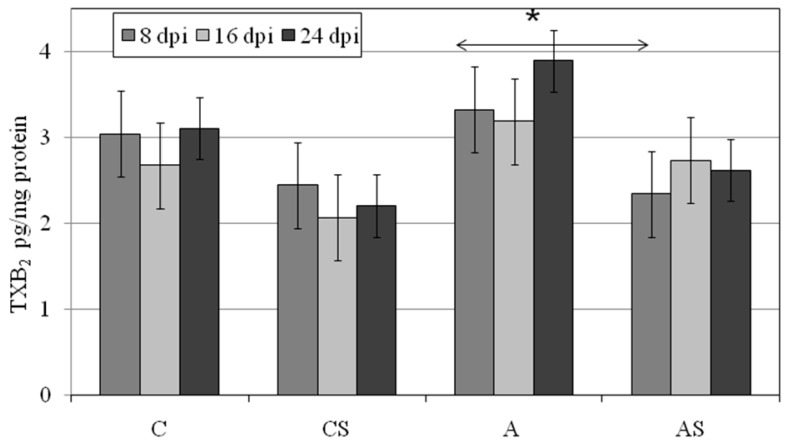
Changes in the levels of thromboxane B_2_ (TXB_2_) in the lungs of *Acanthamoeba* sp.-infected mice depending on the host immune status (C, immunocompetent uninfected control group mice; CS, immunosuppressed uninfected mice; A, immunocompetent *Acanthamoeba* sp.-infected mice; AS, immunosuppressed *Acanthamoeba* sp.-infected mice; dpi days post infection); Data represent the ± standard deviation (SD) for six in dependent experiments * *p* ≤ 0.05 for the significance of difference A vs. AS (Mann–Whitney *U* test).

In the lungs of *Acanthamoeba* sp.-infected immunocompetent mice TXB_2_ at 16 and 24 dpi was higher than in control immunocompetent group at the same times, but not statistically significantly. In immunosuppressed animals: AS and CS groups lung TXB_2_ was the highest at 16 and 8 dpi, respectively. In the CS group, TXB_2_ could be arranged in the following descending order: 8 > 24 > 16 dpi, and in the AS group a reverse dependence was observed (16 > 24 > 8 dpi), although these differences were statistically significant (Figure 5). In the lung of *Acanthamoeba* sp.-infected immunocompetent mice TXB_2_ was statistically significantly higher at 8 dpi than in infected immunosuppressed animals (*p* = 0.03).

### 2.6. Immunohistochemistry

**Figure 6:**
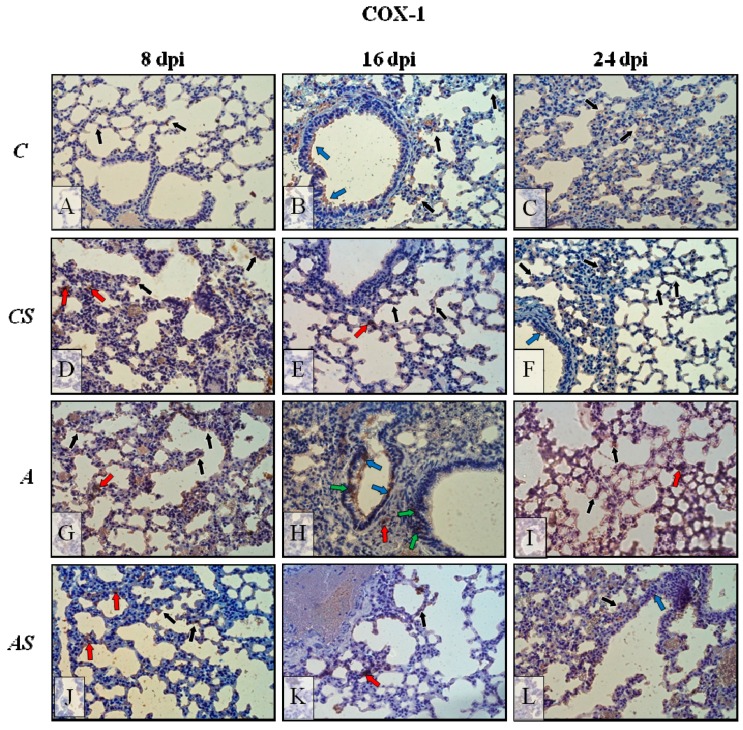
Representative microphotography showing immunoexpression (brown color) of COX-1 in mice lungs from control group (**A**–**C**), control drug treated group: CS (**D**–**F**), infected by *Acanthamoeba* sp. (**G**–**I**) and treated by drug during infection groups (**J**–**L**) in 8, 16 and 24 days post infection (dpi). The examples of immunopositive cells: epithelial cells of bronchioles, blue arrows; pneumocytes, black arrows; stromal cells, stromal cells of lung parenchyma—red arrows, intraepithelial lymphocytes—green arrows ×40. (C, immunocompetent uninfected control group mice; CS, immunosuppressed uninfected mice; A, immunocompetent *Acanthamoeba* sp.-infected mice; AS, immunosuppressed *Acanthamoeba* sp.-infected mice; dpi, days post infection).

**Figure 7:**
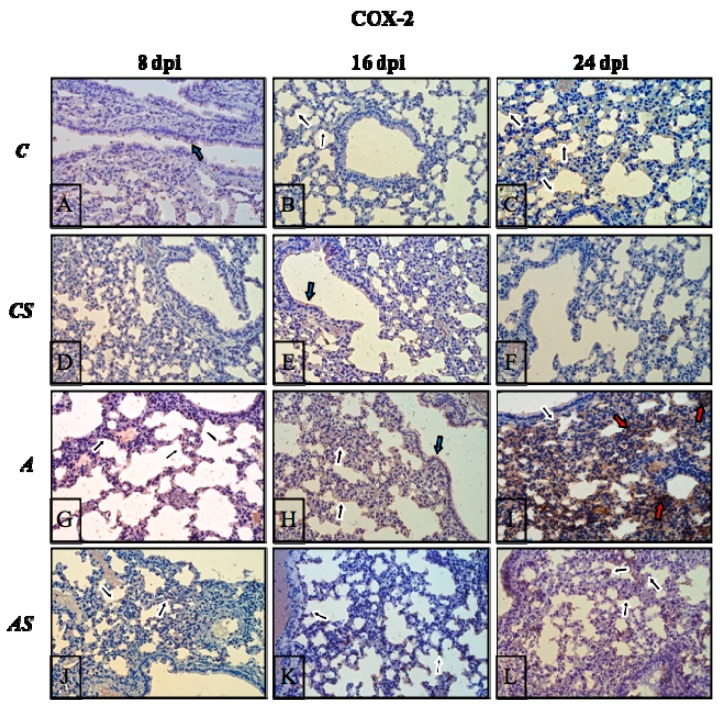
Representative microphotography showing immunoexpression (brown color) of COX-2 in mice lungs from control group (**A**–**C**), control drug treated group (**D**–**F**), infected by *Acanthamoeba* sp. (**G**–**I**) and treated by drug during infection groups (**J**–**L**) in 8, 16 and 24 days post infection (dpi). The examples of immunopositive cells: epithelial cells of bronchioles—blue arrows; pneumocytes—black arrows; stromal cells of lung parenchyma—red arrows. Objective magnification: ×40. (C, immunocompetent uninfected control group mice; CS, immunosuppressed uninfected mice; A, immunocompetent *Acanthamoeba* sp.-infected mice; AS, immunosuppressed *Acanthamoeba* sp.-infected mice; dpi, days post infection).

The results of the immunohistochemical reactions showed that in *Acanthamoeba*-infected immunosuppressed mice during infection, the lungs exhibited changes in COX-1 (Figure 6G–L, respectively) and COX-2 (Figure 7G–L, respectively) expression in comparison to the control group (Figure 6 and Figure 7A–C) and the uninfected immunosuppressed mice (Figure 6 and Figure 7D–F).

In control mice lungs (Figure 6A–C), immunoexpression of COX-1 was low, almost at the same level regardless of the day of the infection (8, 16 and 24), with the enzyme visible mainly in pneumocytes and bronchial epithelial cells (black and blue arrows, respectively). After immunosuppression of the control animals, immunoexpression of COX-1 appears to be slightly higher (Figure 6D–F) and situated mostly in pneumocyte cytoplasm (black arrows), also in the apical region of epithelial cells bronchioles (but at a very low intensity; blue arrows) and sporadically in interstitial cells (red arrows). After *Acanthamoeba* sp. infection of immunocompetent hosts the level of COX-1 (Figure 6G–I) increased and intensified during the days following infection; COX-1-positive cells were pneumocytes (black arrows), bronchial epithelial cells (blue arrows), parenchyma cells (red arrows) and also intraepithelial lymphocytes localized in lining epithelium of bronchioles (green arrows). In immunosuppressed *Acanthamoeba* sp.-infected mice a decrease in COX-1 detection was observed (Figure 6J–L) in comparison to immunocompetent *Acanthamoeba* sp.-infected mice (Figure 6G–I) and was about the same level as in control groups (Figure 6A–F), but still at 24 dpi immunoreactivity was higher than in the previous days of infection in this group (AS). Extinction/blanking of immunoreactivity mainly concerned bronchial epithelial and parenchyma cells (but a few immunopositive cells were visible; red arrows), pneumocytes were constantly showing COX-1 presence, but at a lower level.

In both uninfected groups (C, CS), the immunoexpression of COX-2 was very low at almost imperceptible levels (Figure 7A–F). Among the COX-2-positive cells were pneumocytes and epithelial cells (their apical part/cilia) of bronchioles (black and blue arrows). Decisive elevations in COX-2 level were observed in the lungs of the infected groups of mice (Figure 7G–I), wherethe highest expression of this isoform of cyclooxygenase was noted at 24 dpi (Figure 7I); where besides pneumocytes (black arrows) or poorly positive bronchial epithelial cells (blue arrows), strong immunopositive levels were also noted in parenchyma cells (red arrows). The drug treatment resulted in a decrease of immunoreactivity of lung tissue (especially at 8 dpi) (Figure 7J), after that, it started to increase again (Figure 7K,L), but nevertheless, was at a lower level than in the infected immunocompetent animals (Figure 7G–I); among the mice of group (AS), the presence of COX-2 was noted most of all in the epithelial cells of alveoli (black arrows).

## 3. Discussion

Pulmonary epithelial cells play an active role in inflammation by producing cytokines and eicosanoids, which modulate the inflammatory and immunological host responses [19,31,32]. Little is known about the expression and activity of inflammatory enzymes COX-1 and COX-2 and their products in lungs infected by parasites, including free-living amoeba.

This study demonstrated that *Acanthamoeba* sp. strain (AM 22) isolated from the airways of a patient with AML and atypical pneumonia symptoms in our previous research was pneumophilic in both immunocompetent and immunosuppressed mice [2]. AM 22 significantly reduced total body mass in both immunocompetent and immunosuppressed hosts at 16 dpi. A reduction in body weight in immunocompetent amoeba-infected mice was not surprising, as confirmed in other studies including experimentally inducted cerebral malaria, blastocystosis and opportunistic *Cryptosporidium* sp. infection [33,34,35]. Moreover, infection with *Acanthamoeba* sp. did not affect lung weight in immunocompetent hosts and immunological host status did not significantly alter the weight ratio of lungs during the infection. An increase in lung weight is due primarily to the influx of inflammatory cells, as suggested by Wilson et al. [36].

For the first time, we observed that *Acanthamoeba* sp. induced a strong expression of COX-1 and COX-2 proteins in the lungs of immunocompetent hosts throughout infection. Increased COX-1 expression could be expected as it is constitutively expressed in most cells and tissues, and also involved in inflammatory processes, including lung inflammation [37]. Some researchers suggest that *Acanthamoeba* sp. elicits allergic airway symptoms in mice, so it is probable that this parasite may be one of the triggers of human airway allergic inflammation [4]. COX-1 plays a critical role in regulating airway function and airway inflammation following an allergic stimulus, but in some cases, such as lung tumors, the development of inflammation may result in increased expression of COX-1 gene and its protein expression [37,38]. We observed that COX-1 expression in the lungs from immunocompetent *Acanthamoeba* sp.-infected mice correlated with the infection time and that the concentrations of PGE_2_ and TXB_2_ in the lungs increased (but not significantly) over the following days of infection.

COX-2 has complex and poorly understood roles in anti-pathogen immunity [39]. The induction of COX-2 expression in lung tissues may be related to the direct regulatory function of pulmonary epithelial cells via cytokines released from alveolar macrophages on specific epithelial cells receptors, e.g., IL-β, a potent pro-inflammatory cytokine that is crucial for host-defense response [40]. In pulmonary cryptosporidiosis and in virus lung invasions, stimulation of COX-2 production is dependent on nuclear transcription factor (NF-κB), which plays a role in immune and inflammatory processes [22,41,42]. Probably, *Acanthamoeba* sp. in the lung might cause marked upregulation of NF-κB, enhanced binding of NF-κB to COX-2 promoter, and COX-2 mRNA expression, then COX-2 protein production [22,37]. It is possible that *Acanthamoeba* sp. in the lungs, similar to other pulmonary pathogens, stimulates the expression of a number of proinflammatory gene products, including COX-2 and inducible nitric oxide synthase (iNOS) [22,42]. It has also been observed that COX-2 is regulated by Toll-like receptor 4 (TLR-4) in intestinal cells, and that TLR-4-mediated signaling is responsible for mucosal COX-2 expression and PGE_2_ synthesis in the setting of intestinal inflammation [43,44]. The dependence between COX enzymes and TLR-4 in lung tissues during parasite infection is not clear, albeit Derda et al. [45] found an increased level of expression of TLR2 as well as TLR4 mRNA in lungs during *Acanthamoeba* infection. Moreover, as was suggested by Kosik-Bogacka et al. [21] the increased COX expression and activity in the rat colon and jejunum in intestinal *Hymenolepis diminuta* infection is probably caused by increased levels of free radicals and a weakening of the host’s antioxidant defense induced by the presence of the parasite [21]. In another study, the expression of COX-2 and production of PGE_2_ increased in response to acute respiratory infection with a variety of bacterial organisms and viruses [10]. Szymańsky et al. [10] observed that *Streptococcus pneumonia,* responsible for lungs bacterial invasions, was capable of inducing a strong expression of COX-2 whereas COX-1 was constitutively expressed and remained unaffected in the infected lung. Peres-Buzalaf et al. [46], in experimental bacterial pulmonary tuberculosis, observed ~13-times higher PGE_2_ concentration in the lung at 30 dpi compared to the control group. In contrast, Chen et al. [47] suggested that PGE_2_ is necessary to control *Mycobacterium tuberculosis* during the early stage of invasion. It is suggested that the secretion of this prostaglandin is an element of the feedback loop in the regulation of the immune system [48]. An increase in PGE_2_ suppresses immunity by blocking the activity of the immune system and inhibitsfurther synthesis of PGE_2_ [49]. Vancheri et al. [50] showed that pulmonary PGE_2_ has a role in limitingthe inflammatory response and tissue repair in contrast to its counterparts in other organs. 

A chronic administration of specific COX-1 and COX-2 inhibitors or decreased expression of these enzymes can produce unexpected results. Classic non-steroidal anti-inflammatory drugs (NSAIDs) block prostaglandin synthesis by inhibiting both COX-1 and COX-2 enzymatic activities [51]. Moreover, glucocorticoids strongly suppress the expression of COX-2 induced by inflammatory stimuli [52]. Corticosteroids may modulate COX-2 expression by indirectly reducing IL-4 and IL-13, in contrast to TNF in the asthmatic airway which may induce COX-2 [16]. In this study immunohistochemical reactions showed that the immunosuppression induced by methylprednisolone, a synthetic glucocorticoid with a potent and long-acting anti-inflammatory, antiallergic and immunosuppressive action in *Acanthamoeba* sp.-infected mice caused a decreased in COX-1 detection and decrease in COX-2 immunoreactivity in the lung tissue (especially at the beginning of the infection), but not at 16 dpi and 24 dpi. Hideko Tatakihara et al. [53] in *Trypanosoma cruzi* infection showed that aspirin inhibited COX-1 more than COX-2 and the inhibition was irreversible. Reduced expression of COX-1, together with increased COX-2 expression, was found in the lungs of endotoxin lipopolysaccharide (LPS)-treated rats [54,55]. 

In this study, strong expression of COX-1 and COX-2 proteins in the lungs of immunocompetent hosts induced by the parasite did not result in a significant increase in their products, PGE_2_ and TXB_2_. Inflammation induced by the parasite may have initiated the immune response consisting of the inhibition of the enzyme by its product. The activity of COXs depends on many factors, including those that participate in the initiation and regulation of inflammation, i.e., MAP kinases, such as ERK 1/2, JNK and p38 [56]. PGE_2_ and TXB_2_ produced in cells are released into the extracellular space directly via diffusion or through special membrane transporters-MRP_4_. The released prostaglandin E_2_ influences cells via membrane receptors EP_1_–EP_4_ [57], while thromboxane B_2_ via TP receptors [58]; all of these receptors belong to the G-protein-linked receptor superfamily [57]. Depending on the type of the stimulated receptor, cells experience a change in the levels of intracellular cAMP, calcium ions (Ca^2+^) or change in the activity of phosphoinositide 3-kinase [57,58], which in turn may regulate the activity of COXs and inhibit or activate inflammation.

## 4. Materials and Methods

### 4.1. Acanthamoeba sp. Isolate and Cultivation

The AM 22 strain was isolated from the bronchoaspirate of a 53-year-old man with an acute septic shock. Patient was in acute myeloid leukemia (AML), and atypical pneumonia was diagnosed [2]. The amoebas were grown on agar plates (NN Agar) covered with a suspension of deactivated bacteria *Escherichia coli* (deactivated at 70 °C for 1 h) and incubated at 37 °C for 72 h according to standard methods [59].

### 4.2. Animals and Ethics Statement

The study was conducted on 96 male Balb/c mice obtained from a licensed breeder—the Centre of Experimental Medicine, Medical University in Bialystok, Poland. Animals were about 6–10 weeks old at the beginning of the experiment and their mean weight was 23 g. The mice had genetic and health certificates issued by a veterinarian. This study was approved by the Local Ethics Committee for Experiments on Animals in Szczecin (No. 29/2015, dated 22 June 2015) and Poznań (No. 64/2016 dated 9 September 2016). All animal experiments were conducted and handled in strict accordance with good animal practice within the recommendations in the Guide for the Care and Use of Laboratory Animals. Animals were subject to a 10-min procedure of daily manual handling consisting of keeping the mouse in one hand. Animals were housed in groups of 5 mice per cage on a 12 h light/dark cycle (controlled by automatic timers) in the Animal Facility of the Pomeranian Medical University in Szczecin, and were fed Labofeed H (Morawski, Kcynia, PL0410004p, Poland) and water ad libitum from a stoppered-bottle with a nose-activated nozzle. The room humidity was approximately 56% and air temperature 22 ± 1 °C. To prevent bacterial/viral infections, the cages, feed, water, and bedding were sterilized and changed daily. 

The mice were divided into 4 groups:

- immunocompetent control group (C, *n* = 18) untreated mice.
- immunocompetent (A, *n* = 30) *Acanthamoeba* sp.-infected mice.
- immunosuppressed(AS, *n* = 30) *Acanthamoeba* sp.-infected mice.
- immunosuppressed(CS, *n* = 18) uninfected mice.

### 4.3. Immunosuppression

The Balb/c mice were immunosuppressed by administering 0.22 mg (10 mg/kg) methylprednisolone sodium succinate (MPS, Solu-Medrol, Pfizer, Puurs, Belgium, Europe MA EEIG) in 0.1 mL of 0.9% saline intraperitoneally (i.p.) at −4, −3, −2, −1 and 0 days before inoculation with the amoeba. The drug solution was prepared immediately prior to its administration at a dosage based on literature data [60]. Such an algorithm allowed the development of an experimental model similar to that of immunosuppressed patients. MPS is administered, among others, to patients treated for acute rejection episodes.

### 4.4. Intranasal Inoculation and Pathogenic Test

The mice (groups A and AS) were inoculated intra-nasally with 3 μL of suspension containing 10–20 thousand amoebae. Control animals (groups C and CS) were given the same volume of sterile physiological solution (3 μL of 0.9% NaCl solution). The euthanasia of *Acanthamoeba* sp.-infectedmice were at 8, 16, and 24 days post infection (dpi), depending on clinical signs and degree of infection (excitation or limited mobility, emaciation, ataxia, tremors, changes in behavior, tail chasing, ruffled fur, anorexia, hunched posture, dehydration/reducedskinturgor, not eating or drinking, agony). The animals were sacrificed with a peritoneal overdose of pentobarbital sodium (Euthasol vet, FATRO, Raamsdonksveer, The Netherlands) (2 mL/kg body weight) and subsequently necropsied. The virulence of the amoebae was determined on the degree of infection. Fragments (5 mm × 5 mm) of the lungs were inoculated on NN agar and incubated at 41 °C to assess the infection intensity level [8]. The plates were monitored daily by microscope for 10 days at low magnification. The animals and their lungs were weighed. The relative lung ratios were calculated as follows: lung weight (g)/body weight in infected animals: lung weight (g)/body weight in controls [61]. Pulmonary samples for histological and biochemical analyses were fixed and/or stored in 4% buffered formalin solution (Avantor, Gliwice, Poland), in liquid nitrogen and then stored at −80 °C, respectively.

### 4.5. Clinical Evaluation of the Mice

Infected animals were scored daily for clinical signs, behavior, appetite and mortality. The following elements were analyzed: activity of the mice, feeding, appearance of the fur, hunched position, ataxia and tremors as described in [62]. The points used to assess the clinical status were 0, 1 and 2. The average of three measures was taken, if the average total score was 3, the mice was classified as severely sick (humanitary end point), if between 4 and 7 the mice were moderately sick, and if the total score was over 8, the micewere deemed healthy (without symptoms). 

### 4.6. Western Blotting Analysis of COX-1 and COX-2 Expression

RIPA buffer (pH 7.4) was used for lung sample homogenization using a previously described method [21]. To asses total protein concentrations we used Micro BCA Protein Assay Kits (Thermo Scientific, Rockford, IL, USA). Lung homogenates were subjected to SDS-polyacrylamide gel electrophoresis and examined for protein expression of COX-1 and COX-2. Western blotting analysis of COX-1 and COX-2 expression was performed essentially as previously described by Olszowski et al. [37].

### 4.7. Measurements of Prostaglandin E_2_ and Thromboxane B_2_ Concentrations

The activity of COX-1 and COX-2 enzymes was determined by quantitative measurement of their products: PGE_2_ and TXB_2_ extracted from a homogenate of lung samples using Bakerbond columns (Witko Group, Łódź, Poland). The measurements of PGE_2_ and TXB_2_ levels were conducted using appropriate immunoenzymatic sets (Prostaglandin E_2_ EIA Kit, Cayman, Ann Arbor, MI, USA; Thromboxane B_2_EIA Kit, Cayman, Ann Arbor, MI, USA) according to the manufacturer’s instructions. The concentrations of PGE_2_ and TXB_2_ were expressed in pg per mg protein.

### 4.8. Immunohistochemistry of COX-1 and COX-2 Expression

Paraffin-embedded sections (3–5 μm) of mice lungs were immunostained for the visualization of COX-1 and COX-2 proteins expression. Immunohistochemistry wasperformed using specific primary goat polyclonal antibodies (Santa Cruz Biotechnology, Dallas, TX, USA) against COX-1 and COX-2 in a final 1:100 dilution. Immunohistochemistry of COX-1 and COX-2 expression were performed essentially as previously described [21].

Firstly, the deparaffinized sections were microwave irradiated in citrate buffer (pH 6.0) to heat induce epitope retrieval. After slow cooling to room temperature, slides were washed in phosphate-buffered saline (PBS) solution twice for 5 min and then incubated for 60 min with primary antibodies. Following this, sections were stained using an avidin-biotin-peroxidase system with diaminobenzidine (DAKO (HRP; Rabbit/Mouse/Goat (DAB+); DakoCytomation, Glostrup, Denmark) as the chromogen, in conformity with the staining procedure instructions included. Sections were washed in distilled H_2_O and counterstained with hematoxylin. For a negative control, specimens were processed in the absence of primary antibodies. Positive staining was defined microscopically (Leica DM5000 B, Hamburg, Germany) by visual identification of brown pigmentation [21].

### 4.9. Statistical Analysis

Statistical analysis was carried using Statistica 10 PL software. The arithmetic means (AM), standard deviations of the AM (SD), medians (Med) were calculated for each studied group. The distribution normality was examined using Shapiro–Wilk *W*-test. The nonparametric tests (Kruskal–Wallis and Mann–Whitney *U* test) were used in the analysis because distributions in most cases deviated from normal. Correlations between the parameters were tested by Spearman rank correlation coefficient (r_s_). The significance level was *p* < 0.05.

## 5. Conclusions

*Acanthamoeba* sp. induced a strong expression of COX-1 and COX-2 proteins in the lungs of immunocompetent mice. These results suggested that similar to COX-2, COX-1 is an important mediator of the pathophysiology in experimental acanthamoebiasis. In this study, we noted that strong expression of COX-1 and COX-2 proteins in the lungs of immunocompetent hosts induced by the parasite, does not correspond to significant differences in the expression the eicosanoids PGE_2_ and TXB_2_. Immunosuppression induced by glucocorticoids in *Acanthamoeba* sp.-infected mice caused a decrease in COX-1 and COX-2 (not at the beginning of infection) in lung tissue. We suggest that the signaling pathways important for *Acanthamoeba* sp. induction of lung infection might interact with each other and depend on the host immune status. However, the pathogenesis of pulmonary invasion by *Acanthamoeba* sp. is still poorly understood and documented, and so requires further research.

## Figures and Tables

**Table 1:** Body and lungs weights of male mice (in g) in control and infected groups after 8, 16 and 24 days post *Acanthamoeba* sp. infection (dpi).

Parameter	n/g	Group	C	CS	A	AS	P (K-W Test)
Body mass							
	n		18	18	30	30	
Total	g	AM ± SD	26.30 ± 1.88 *	29.28 ± 1.58	24.57 ± 4.19 *^,#^	28.30 ± 3.29 ^#^	0.0001
		Median	26.59	29.30	25.47	29.30	
		Range	21.34–30.13	27.20–32.80	13.41–29.50	20.04–33.01	
	n		6	6	10	10	
8 dpi	g	AM ± SD	25.47 ± 2.92	28.18 ± 0.93	24.15 ± 4.16	29.78 ± 1.25	0.41
		Median	26.06	27.70	25.16	29.45	
		Range	21.34–30.13	27.30–29.05	13.41–27.26	28.41–32.40	
16 dpi	g	AM ± SD	26.55 ± 0.65 *	29.7 ± 1.51	21.65 ± 3.91 *^,#^	28.5 ± 4.45 ^#^	0.001
		Median	26.62	29.70	22.04	29.05	
		Range	25.59–27.55	27.2–31.6	15.58–26.91	20.04–33.01	
24 dpi	g	AM ± SD	26.62 ± 1.60	29.86 ± 1.86	27.92 ± 1.42	28.13 ± 3.43	0.42
		Median	27.06	29.80	28.17	28.90	
		Range	24.14–28.43	27.80–32.80	24.87–29.50	21.40–31.50	
Lung mass							
	n		18	18	30	30	
Total	g	AM ± SD	0.19 ± 0.03 *	0.22 ± 0.03	0.21 ± 0.18 *	0.20 ± 0.04	0.001
		Median	0.19	0.20	0.17	0.20	
		Range	0.15–0.23	0.10–0.30	0.12–1.10	0.10–0.30	
	n		6	6	10	10	
8 dpi	g	AM ± SD	0.20 ± 0.04	0.22 ± 0.04	0.18 ± 0.05	0.21 ± 0.03	0.25
		Median	0.21	0.20	0.17	0.20	
		Range	0.15–0.26	0.20–0.30	0.12–0.28	0.20–0.30	
16 dpi	g	AM ± SD	0.19 ± 0.02	0.23 ± 0.05	0.16 ± 0.03	0.2 ± 0.05	0.42
		Median	0.19	0.20	0.16	0.20	
		Range	0.17–0.22	0.2–0.30	0.12–0.21	0.1–0.30	
24 dpi	g	AM ± SD	0.19 ± 0.03	0.20 ± 0.07	0.28 ± 0.29	0.20 ± 0.05	0.21
		Median	0.19	0.20	0.19	0.20	
		Range	0.15–0.23	0.10–0.30	0.13–1.10	0.10–0.30	

C, immunocompetent uninfected control group mice; CS, immunosuppressed uninfected mice; A, immunocompetent *Acanthamoeba* sp.-infected mice; AS, immunosuppressed *Acanthamoeba* sp.-infected mice; AM arithmetic mean; SD standard deviation; P level of significance; K-W test Kruskal-Wallis test; * *p* ≤ 0.05 for the significance of difference vs. control (Mann–Whitney *U* test); ^#^ *p* ≤ 0.05 for the significance of difference A vs. AS (Mann–Whitney *U* test).

## Abbreviations

A	immunocompetent *Acanthamoeba* sp.-infected mice
AK	*Acanthamoeba* keratitis
AM	arithmetic mean
AM 22	amoebic strain no. 22
Med	medians
AML	acute myeloid leukemia
AS	immunosuppressed *Acanthamoeba* sp.-infected mice
C	immunocompetent uninfected control group mice
CA	cutaneous acanthamoebiasis
cAMP	cyclic adenosine monophosphate
COX-1	cyclooxygenase-1
COX-2	cyclooxygenase-2
CS	immunosuppressed uninfected mice
dpi	days post infection
ERK 1/2	extracellular signal-regulated kinase
PGA	prostaglandin
PGE_2_	prostaglandin E_2_
GAE	granulomatous amebic encephalitis
IK	immunocompetent
IL-4	interkeukin 4
IL-13	Interleukin 13
IL-β	interleukin 1 beta
iNOS	inducible nitric oxide synthase
IS	immunosuppressed
JNK	c-Jun N-terminal kinases
K-W test	Kruskal-Wallis test
LI	relative weight ratio of lungs
LPS	lipopolysaccharide
MPS	methylprednisolone sodium succinate
MRP_4_	multidrug resistance protein 4
NF-κB	nuclear transcription factor
NSAIDs	non-steroidal anti-inflammatory drugs
p38	mitogen-activated protein kinase
r_s_	correlation coefficient
SD	standard deviation
TLR2	Toll-like receptor 2
TLR4	Toll-like receptor 4
TXs	thromboxanes
TXA_2_	thromboxane A_2_
TXB_2_	thromboxane B_2_
